# Transplantation of Human Neural Stem Cells in a Parkinsonian Model Exerts Neuroprotection via Regulation of the Host Microenvironment

**DOI:** 10.3390/ijms161125966

**Published:** 2015-11-05

**Authors:** Fu-Xing Zuo, Xin-Jie Bao, Xi-Cai Sun, Jun Wu, Qing-Ran Bai, Guo Chen, Xue-Yuan Li, Qiang-Yi Zhou, Yuan-Fan Yang, Qin Shen, Ren-Zhi Wang

**Affiliations:** 1 Department of Neurosurgery, Peking Union Medical College Hospital, Chinese Academy of Medical Sciences & Peking Union Medical College, Beijing 100730, China; zfx819_1986@163.com (F.-X.Z.); xinjieabao@163.com (X.-J.B.); lixueyuan-82@163.com (X.-Y.L.); zhouqy7019@hotmail.com (Q.-Y.Z.); yangyf06@mails.tsinghua.edu.cn (Y.-F.Y.); 2 Center for Stem Cell Biology & Regenerative Medicine, Center for Life Sciences, School of Medicine, Tsinghua University, Beijing 100084, China; sun_xicai@126.com (X.-C.S.); jwu11@mails.tsinghua.edu.cn (J.W.); biowork@foxmail.com (Q.-R.B.); happycg2009@163.com (G.C.)

**Keywords:** Parkinson’s disease, neural stem cells, transplantation, niche, endogenous de-differentiated astrocytes

## Abstract

Parkinson’s disease (PD) is characterized by a progressive loss of dopaminergic neurons and consequent dopamine (DA) deficit, and current treatment still remains a challenge. Although neural stem cells (NSCs) have been evaluated as appealing graft sources, mechanisms underlying the beneficial phenomena are not well understood. Here, we investigate whether human NSCs (hNSCs) transplantation could provide neuroprotection against DA depletion by recruiting endogenous cells to establish a favorable niche. Adult mice subjected to 1-methyl-4-phenyl-1,2,3,6-tetrahydropyridine (MPTP) were transplanted with hNSCs or vehicle into the striatum. Behavioral and histological analyses demonstrated significant neurorescue response observed in hNSCs-treated animals compared with the control mice. In transplanted animals, grafted cells survived, proliferated, and migrated within the astrocytic scaffold. Notably, more local astrocytes underwent de-differentiation, acquiring the properties of NSCs or neural precursor cells (NPCs) in mice given hNSCs. Additionally, we also detected significantly higher expression of host-derived growth factors in hNSCs-transplanted mice compared with the control animals, together with inhibition of local microglia and proinflammatory cytokines. Overall, our results indicate that hNSCs transplantation exerts neuroprotection in MPTP-insulted mice via regulating the host niche. Harnessing synergistic interaction between the grafts and host cells may help optimize cell-based therapies for PD.

## 1. Introduction

A major hallmark of Parkinson’s disease (PD) is an extensive loss of dopaminergic neurons in the substantia nigra (SN) and consequent dopamine (DA) deficit in the striatum [1]. Although symptomatic improvements can be achieved by systemic administration of L-dopa or DA agonists, there are still a great number of challenges such as diminished effectiveness and considerable side effects [2].

Alternatively, cell replacement therapies in PD are based on the premise that grafts can restore dopaminergic neurotransmission, providing a functionally efficient substitute for neuron loss [3]. Neural stem cells (NSCs) are defined as multipotent, self-renewing progenitors [4,5], possessing intrinsic capacity to rescue dysfunctional neural pathways. NSCs have been evaluated as appealing donor graft sources [6,7,8], since they are endowed with certain differentiation stages [9]. Nonetheless, several disadvantages have prevented its meaningful applicability [6,8,9]: (i) limited supply of cell sources with homologous species; (ii) unstable cell survival and slow maturation; (iii) minimal differentiation into dopaminergic neurons.

Recently, more studies have focused on the host microenvironment or the “niche”, which is critical to determine the fate of donor cells [10,11]. Particularly, astrocytes are the support cells of the nervous system, and host-derived reactive astrocytes in response to brain insult largely comprise the inflammatory niche. Firstly, the astrocytes endfeet wrap brain blood vessels to establish the gliovascular interface [12]. These cells participate in blood flow regulation, nutrient transport, and modulation of synaptic transmission [12,13]. Indication of migratory patterns of endogenous NSCs or neural precursor cells (NPCs) highlights the important role of astrocytes as host scaffold [14], and raises the question of whether grafted cells themselves may attract local astrocytes, which in turn facilitate survival and migration of the xenografts; Secondly, reactive astrocytes de-differentiate into a phenotype resembling radial glial cells (RGCs) following DA depletion [4,15,16,17], and have been proposed as the resident adult NSCs/NPCs which might exert beneficial effects. Several findings have provided evidence that reactive astrocytes can be reprogrammed into immature [18] or mature neurons [19] by transcription factors. In light of these studies, recruitment of endogenous de-differentiated astrocytes (EDAs) residing within the brain niche might be promising for PD therapies, because they might exhibit a remarkable capacity for differentiating into neuronal phenotypes. However, there have been few detailed reports concerning the glial response to NSC transplantation in PD; Thirdly, previous evidence has suggested that astrocytes, particularly the EDAs are central to endogenous neuroprotection by release of neurotrophic factors such as glial cell line-derived neurotrophic factor (GDNF), brain-derived neurotrophic factor (BDNF), insulin-like growth factor-1 (IGF-1), neurotrophin-3 (NT-3), and epidermal growth factor (EGF), *etc*. [20,21,22]. Therefore, investigation of the beneficial effects, particularly the capability of producing growth factors by recruited endogenous NSCs/NPCs (e.g., EDAs) following transplantation would be crucial for increasing accessibility of the niche. In addition to the above-mentioned elements, activated microglia, the feature of neuroinflammation, are detrimental to the host and grafted cells via releasing multiple cytotoxic molecules in the nigrostriatal pathway in PD [23,24]. Inhibition of microglia and proinflammatory cytokines appears to protect dopaminergic neurons and ameliorate behavioral disabilities.

In our study, adult mice were subjected to 1-methyl-4-phenyl-1,2,3,6-tetrahydropyridine (MPTP) lesions followed by intrastriatal transplantation of human neural stem cells (hNSCs). Initially, we assessed whether hNSCs could produce behavioral benefits and protect against DA depletion. We found that hNSCs transplantation not only produced immature neurons that survived and migrated within the astrocytic scaffold, but also promoted de-differentiation of local astrocytes, production of neurotrophic factors, and inhibition of microglia as well as proinflammatory cytokines, demonstrating that hNSCs transplantation could provide neuroprotection via regulating the host niche. Better understanding of the underlying mechanisms will facilitate application of hNSCs therapy to the clinic.

## 2. Results

### 2.1. Behavioral Tests

**Figure 1:**
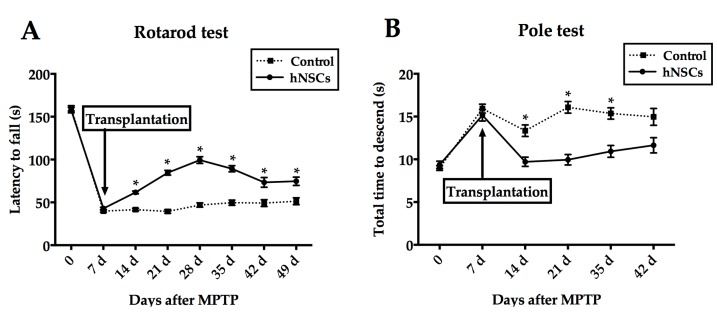
Transplantation of hNSCs (human neural stem cells) promotes functional recovery following MPTP injection. Motor performance in rotarod (**A**) and pole (**B**) tests of the hNSCs-treated or control groups demonstrated significant differences starting at 14 days after MPTP. Values represent mean ± SEM (* *p* < 0.05; two-way ANOVA). hNSCs, human neural stem cells; MPTP, 1-methyl-4-phenyl-1,2,3,6-tetrahydropyridine.

The rotarod as well as pole test were compared in different groups. No difference was observed before cell transplantation. However, significant improvement in duration on the rotarod appeared in grafted mice starting from seven days after hNSCs transplantation compared with controls (*p* < 0.05, Figure 1A). At 26 rpm, hNSCs-treated mice remained on the rotarod significantly longer than the control animals. Interestingly, the duration decreased from 28 days after treatment (still statistically significant compared with control) as depicted in Figure 1A. For the pole test, hNSCs-treated mice took a significantly shorter time to complete the paradigm after seven days post-transplantation except for the time point of 42-days (Figure 1B).

### 2.2. hNSC (Human Neural Stem Cells) Transplantation Protects both Cell Bodies and Axons of the Nigrostriatal Dopaminergic Pathway

**Figure 2:**
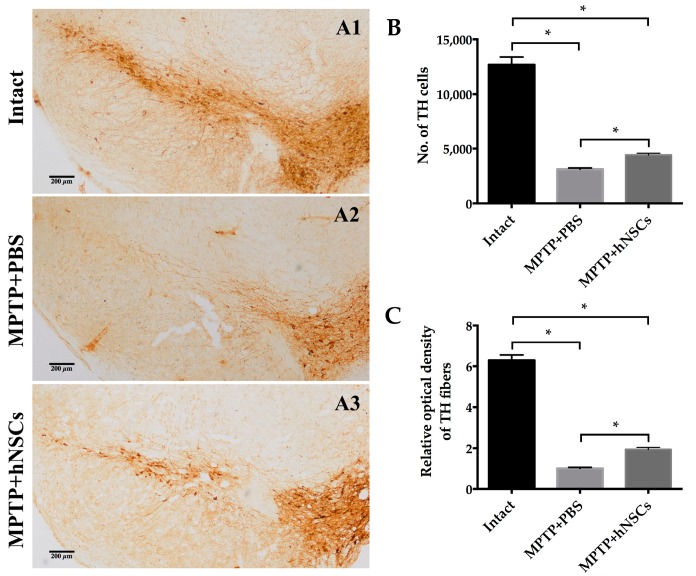
The hNSCs-treated mice are more resistant against MPTP neurotoxicity. (**A**) Although the overall number of dopaminergic neurons in hNSCs-treated mice (**A3**) was still smaller than that of cells in intact animals without MPTP (absolute controls) (**A1**), significantly more remaining TH cells were observed in transplanted mice (**A3**) compared with animals given PBS (**A2**); Quantification of nigral TH positive neurons (**B**) and optical density of striatal TH positive fibers (**C**) revealed significant recovery in hNSCs-treated mice compared with animals given PBS. Data of optical densities are normalized to the mean of PBS-treated animals. Scale bars represent 200 μm. Bars represent mean ± SEM (* *p* < 0.05; two-tailed Student’s *t*-test). TH, tyrosine hydroxylase; SN, substantia nigra; PBS, phosphate buffered saline.

To assess effects of nigrostriatal protection, we examined the optical densities of dopaminergic axons in the striatum and stereologically counted the number of dopaminergic neurons in the SN stained for tyrosine hydroxylase (TH). At 42 days following hNSCs transplantation, there was substantial restoration of innervation (Figure 2C). Values were normalized to the mean of mice given 0.1 M phosphate buffered saline (PBS). Furthermore, hNSCs-transplanted mice had an average of 4423.53 ± 146.00 cells expressing TH in the SN when compared with vehicle-infused animals which had only 3116.89 ± 119.20 dopaminergic neurons (*p* < 0.05, Figure 2B).

### 2.3. Survival, Migration and Phenotypic Fate of Grafted hNSCs

**Figure 3:**
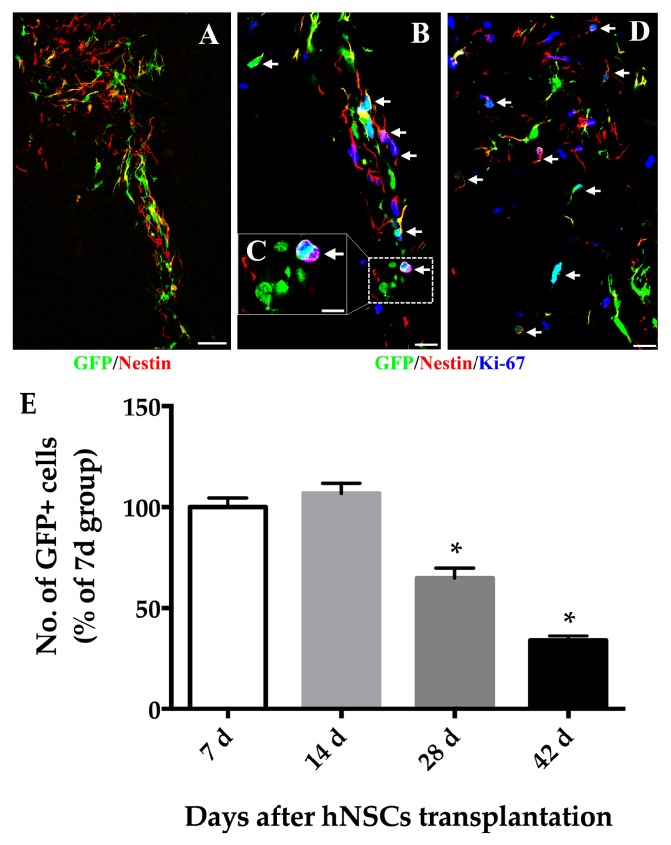
The hNSCs express the marker of neural precursor cell and proliferate at an early stage following transplantation. Immunofluorescence staining showed that a large number of GFP positive hNSCs (**A**–**D**; **green**) expressed Nestin (**A**–**D**; **red**), some of which co-labeled with Ki-67 (**B**–**D**, **blue**). At 7 days post-transplantation (**B**), the hNSCs dispersed along the grafted core which accommodated some of GFP/Nestin/Ki-67 positive cells (arrows); (**C**) Higher magnification images of the boxed areas in (**B**) demonstrated one representative proliferating stem cell (arrow) with enlarged double nuclei; (**D**) At 14 days post-transplantation, some grafted cells still expressed Nestin, which demonstrated that they were at the early stage of neurogenesis and remained poorly differentiated. Scale bars represent 50 μm in (**A**); 20 μm in (**B**,**D**); 10 μm in (**C**); (**E**) Grafted cells survived well for at least 14 days, but significantly fewer cells survived 28 and 42 days following treatment. Cell number was expressed as percentage of day-7 group. Values represent mean ± SEM (* *p* < 0.05; two-tailed Student’s *t*-test). GFP, green fluorescent protein.

Two weeks after hNSCs transplantation, we analyzed grafted cell survival and migration in the striatum. Grafted cells in treated mice appeared to live with normal morphologies. The overall number surviving in hNSCs transplants at day-14 appeared to be greater than those at day-7, although this failed to achieve statistical significance (*p* > 0.05). The number of surviving cells was estimated to be more than that of actually transplanted because cells within the transplants continued to proliferate. Approximately, 68.09 ± 3.08 percent of grafted cells expressed Ki-67 at day-7 (Figure 3B). However, the number of transplanted cells present in the host brain gradually decreased after longer time (by 28 and 42 days following transplantation, 64.79 ± 4.89 and 33.91 ± 2.26 percent of grafts at day-7 respectively) (Figure 3E).

**Figure 4:**
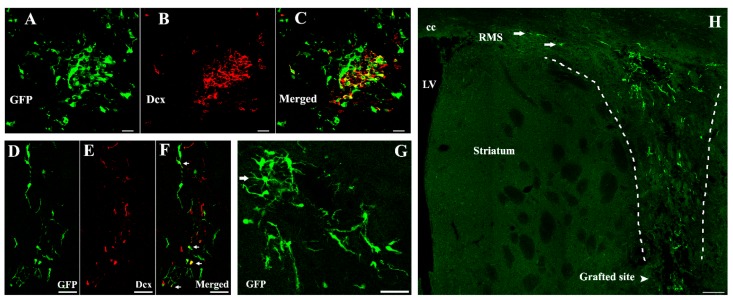
hNSCs differentiate into immature neurons and migrate within the host striatum. Typically, these GFP positive cells (**A**,**C**; **green**) were immunoreactive to Dcx (**B**,**C**; **red**) 14 days post-transplantation (54.49% ± 3.15%). Additionally, arrows in (**F**) illustrated donor-derived immature neurons within the migratory chains. Transplanted hNSCs dispersed along the dotted lines in (**H**) and migrated towards the cc or even connected with the RMS (arrows) 14 days after transplantation. Some of the GFP positive cells extended their neurites, pointing in the direction of migration at this time point, while arrow in (**G**) demonstrated that transplanted hNSCs exhibited mature appearance with elongated shape and numerous processes 21 days following treatment. Scale bars represent 20 μm in (**A**–**C**); 50 μm in (**D**–**G**); 100 μm in (**H**). GFP, green fluorescent protein; Dcx, doublecortin; LV, lateral ventricle; cc, corpus callosum; RMS, rostral migratory stream.

In terms of migration of grafted cells, hNSCs seemed to be broadly distributed around the transplanted core in the striatum (Figure 4). Many of the migrating cells expressed nestin, some of which co-labeled with Ki-67 (Figure 3). At 14 days post-treatment, grafted cells mainly dispersed along the grafted trajectory and migrated towards the corpus callosum (cc) or even connected with the rostral migratory stream (RMS) (Figure 4H). Typically, these cells were of an elongated shape, which sprouted and extended their neurites, pointing in the direction of migration. Grafts were strongly immunoreactive to doublecortin (Dcx) (54.49% ± 3.15% of green fluorescent protein (GFP) positive cells) accompanying cell migration since 14 days post-treatment (Figure 4). However, no astrocytic differentiation was observed because grafted cells did not stain for glial fibrillary acidic protein (GFAP) at all time points.

### 2.4. Reactive Astrocytes Response to hNSCs Transplantation

**Figure 5:**
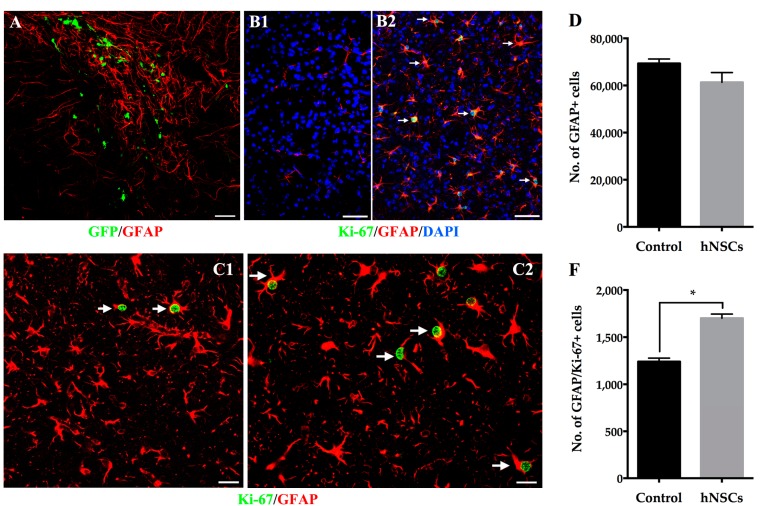
Striatal astrocytes in response to hNSCs transplantation. (**A**) Local GFAP positive astrocytes (**red**) accumulated around the xenografts (**GFP**, **green**) when the hNSCs continued to migrate within the striatum 14 days following treatment. Most of the astrocytes (**B2**, **red**) co-localized with Ki-67 (**B2**, **green**), indicating that they became reactive and re-exhibited proliferative capacity, whereas few astrocytes could be found in the striatum of intact animal without MPTP (**B1**). Cell nuclei were counterstained with DAPI (**blue**). Moreover, the astrocytes displayed hypertrophic cell bodies with thick processes (**arrows in C**). More Ki-67 positive astrocytes were observed in the striatum of mice receiving hNSCs (**C2**) compared with animals given vehicle (**C1**) 14 days after transplantation. Although no significant difference of GFAP positive cell number was observed in hNSCs-treated mice when compared with animals given vehicle (**D**), there was a greater increase of Ki-67/GFAP positive cells in the striatum of treated mice (**F**). Data are expressed as mean ± SEM (* *p* < 0.05; two-tailed Student’s *t*-test). Scale bars represent 50 μm in (**A**,**B**); 20 μm in (**C**). GFAP, glial fibrillary acidic protein. DAPI, 4′,6-diamidino-2-phenylindole.

**Figure 6:**
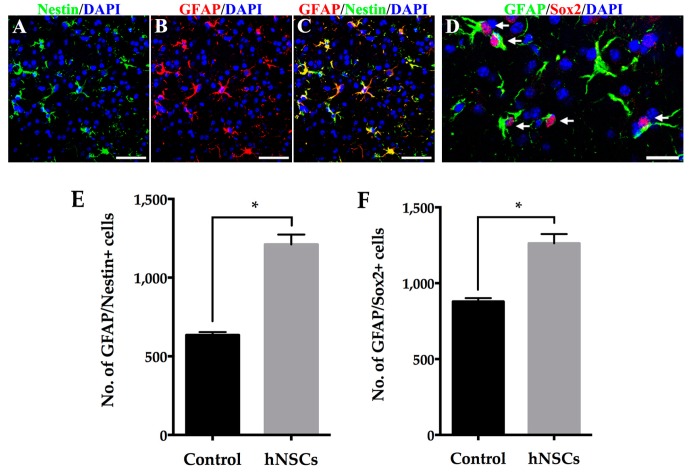
hNSCs transplantation promote de-differentiation of host-derived astrocytes in the striatum. A large number of GFAP positive astrocytes (**B**,**C**, **red**) expressed intermediate filament Nestin (**A**,**C**, **green**). Furthermore, most local astrocytes (**arrows in D**, **green**) co-localized with transcription factor Sox2 (**arrows in D**, **red**), suggesting that they de-differentiated into a phenotype resembling RGCs that were shown to be transient NPCs. Cell nuclei were counterstained with DAPI (**blue**) (**A**–**D**); Quantification of GFAP/Nestin positive cells (**E**) and GFAP/Sox2 positive cells (**F**) in the striatum showed significantly more EDAs in hNSCs-treated mice compared with the control animals. Bars represent mean ± SEM (* *p* < 0.05; two-tailed Student’s *t*-test). Scale bars represent 50 μm in (**A**–**C**); 20 μm in (**D**). RGCs, radial glial cells; NPCs, neural precursor cells; EDAs, endogenous de-differentiated astrocytes.

hNSCs transplantation did not cause a statistically significant difference in the presence of host-derived GFAP positive astrocytes compared with that in the animals given vehicle. However, significantly greater increase of Ki-67 positive astrocytes was found in the striatum of mice receiving hNSCs (Figure 5C). Furthermore, astrocytes mainly accumulated along the grafted trajectory within the hNSCs (Figure 5A), most of which displayed hypertrophic cell bodies extending thick process (Figure 5B2,C). It is noteworthy that, there were a great number of GFAP/Nestin and GFAP/Sox2 positive cells in the striatum, suggesting these GFAP positive astrocytes were in the process of de-differentiation, acquiring the properties of NSCs/NPCs (Figure 6). Quantification showed significantly more EDAs in hNSCs-treated mice in comparison with the control animals.

### 2.5. Quantitative Analysis of Neurotrophic Factors

**Figure 7:**
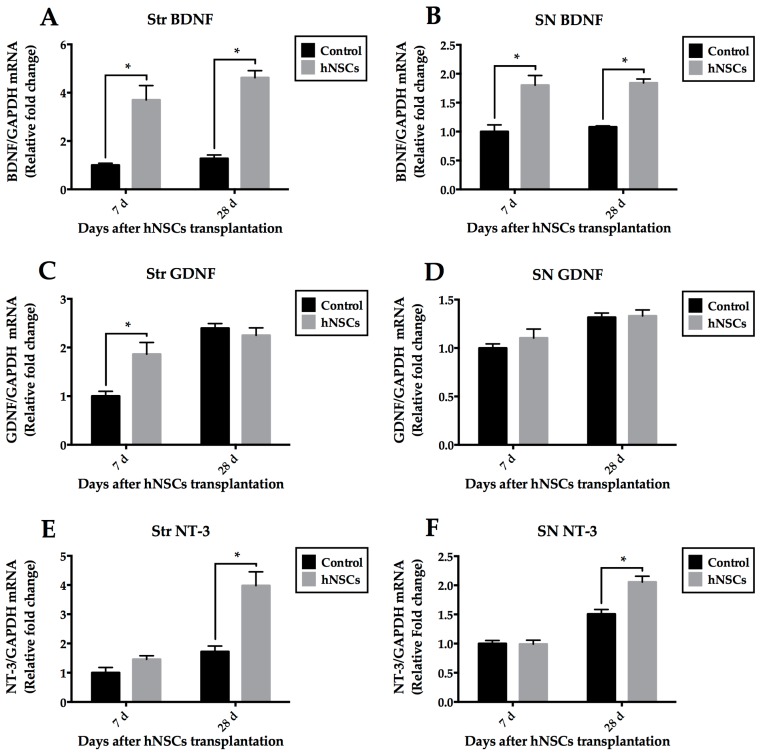
QRT-PCR analysis of host-derived BDNF, GDNF, and NT-3 mRNA levels in different tissues (Str and SN). BDNF was significantly up-regulated at each time point in mice receiving hNSCs (**A**,**B**); The effect was also seen in the treated mice with a trend towards greater increase of NT-3 mRNA levels compared with control mice, but it did not achieve significant difference at seven days (**E**,**F**); No difference of GDNF levels was observed at each time point except for expression at seven days in the striatum (**C**,**D**). All results were normalized to GAPDH mRNA levels and expressed as relative fold change to the levels at 7-day of the control group. Data are expressed as mean ± SEM (* *p* < 0.05; two-way ANOVA). QRT-PCR, real time quantitative reverse transcriptase polymerase chain reaction; BDNF, brain-derived neurotrophic factor; GDNF, glial cell line-derived neurotrophic factor; NT-3, neurotrophin-3; Str, striatum; SN, substantia nigra; GAPDH, Glyceraldehyde 3-phosphate dehydrogenase.

To test the possibility that transplanted hNSCs stimulate the host cells to provide a favorable niche, we examined the mRNA levels of neurotrophic factors, using primers specifically recognizing mouse but not human GDNF, BDNF, and NT-3. Total RNA was extracted from striatal and nigral tissues at early (7-day) and later (28-day) stages. Real time quantitative reverse transcriptase polymerase chain reaction (QRT-PCR) analysis showed significant increases in BDNF mRNA levels of the hNSCs treated group at each time point (Figure 7A,B). The effect was also seen in the treated mice with a trend towards greater increase of NT-3 mRNA levels compared to the control mice, but it did not achieve statistical difference at seven days (Figure 7E,F). No difference of GDNF levels was observed at each time point except for expression in the tissue of striatum at seven days (Figure 7C,D). All results were normalized to mRNA levels of glyceraldehyde 3-phosphate dehydrogenase (GAPDH) and showed as relative expression to the control levels at seven days.

**Figure 8:**
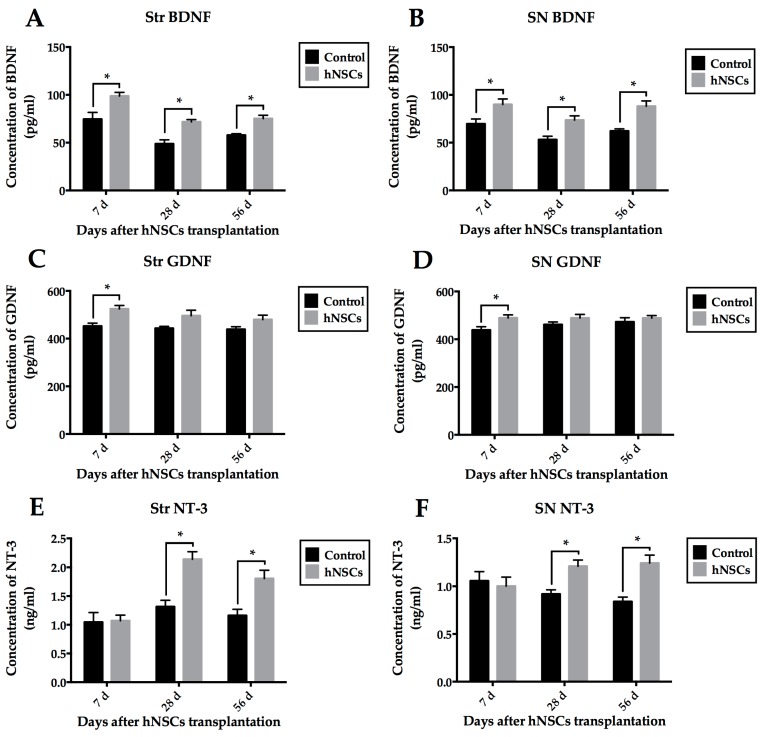
Detection of BDNF, GDNF, and NT-3 protein levels in different tissues (Str and SN) by ELISA. Transplantation of hNSCs induced significant increases in BDNF (**A**,**B**) and NT-3 (**E**,**F**) protein expression, when compared with vehicle-treated control group. Although hNSCs treatment significantly induced production of GDNF at seven days, compared with control group, there was no difference of GDNF protein level at later stages (**C**,**D**). Data are displayed as mean ± SEM (* *p* < 0.05; two-way ANOVA). ELISA, enzyme-linked immunosorbent assay.

We also detected mouse protein levels of these growth factors in different tissues at different time points (7-day, 28-day, 56-day) by enzyme-linked immunosorbent assay (ELISA). Transplantation of hNSCs led to statistical increases in BDNF (Figure 8A,B) and NT-3 (Figure 8E,F) protein expression, when compared with those treated with vehicle. Although hNSCs treatment significantly induced production of GDNF at seven days, compared with control group, it failed to result in any difference at later stages (Figure 8C,D).

### 2.6. Inhibition of Microglia and Proinflammatory Cytokines after Transplantation

**Figure 9:**
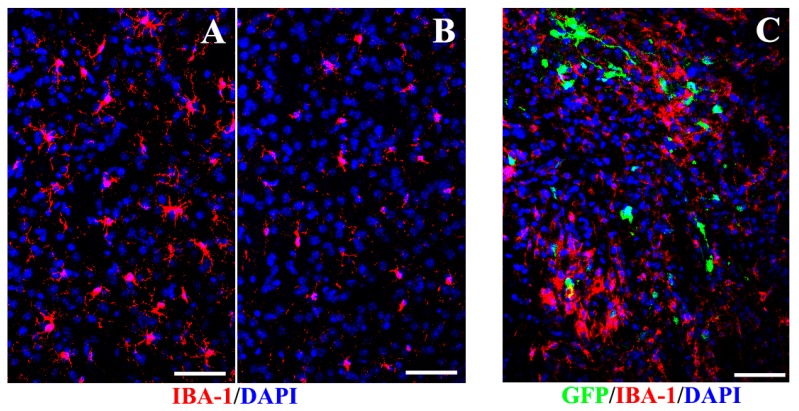

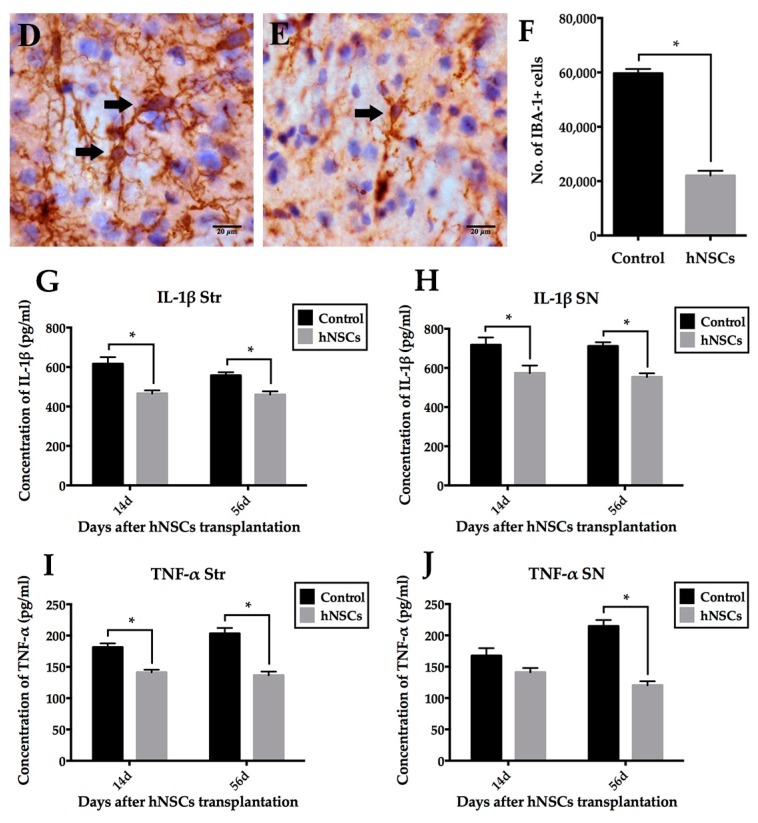
Effects of hNSCs-induced inhibition of host microglia and proinflammatory cytokines. Fewer IBA-1 positive microglia (**red**) presented in the striatum of hNSCs-treated mice (**B**) than the control animals (**A**); (**C**) Transplanted hNSCs (**green**) gathered local microglia with activated forms (**red**) to a limited area around the xenografts. Cell nuclei were counterstained with DAPI (**blue**) (**A**–**C**). Most microglia presented fully activated forms in control mice (**arrows in D**), while arrow in (**E**) indicated the ramified microglia (resting state of microglia) in transplanted animals. Cell nuclei were hematoxylin counterstained in (**D**,**E**). The numbers of microglia in different groups were compared as illustrated in (**F**). Additionally, the expression of IL-1β (**G**,**H**) and TNF-α (**I**,**J**) was downregulated in the striatum and SN of mice receiving hNSCs compared with the control animals. Data are expressed as mean ± SEM (* *p* < 0.05; two-tailed Student’s *t*-test). Scale bars represent 50 μm in (**A**–**C**); 20 μm in (**D**,**E**). IBA-1, ionized calcium-binding adapter molecule 1; IL-1β, interleukin-1β; TNF-α, tumor necrosis factor-α.

Overall, there was a significantly reduced microglial reaction in the striatum of hNSCs-treated mice (30,674.52 ± 2061.42) in contrast to the control (69,372.55 ± 1870.38, mainly presenting activated shape) (*p* < 0.05, Figure 9), suggesting that transplantation exerted a dampening influence on host inflammation. A substantial proportion of ionized calcium-binding adapter molecule 1 (IBA-1) positive microglia presented fully active phagocytic forms in control mice (Figure 9D). The ramified microglia (resting state of microglia) were identified in the surrounding grafted area in animals given hNSCs (Figure 9E). All of the IBA-1 positive microglia were host-derived without expressing GFP. No IBA-1 positive microglia co-labeled with Nestin or Sox2, demonstrating that the reactive cells did not revert to an immature state. Additionally, protein content of IL-1β and TNF-α was downregulated in the striatum and SN of mice receiving hNSCs compared with the control animals (Figure 9G–J).

## 3. Discussion

In the present study, we have demonstrated that transplantation of hNSCs into the striatum of MPTP-insulted mice protected dopaminergic neuron degeneration and improved host neurological function. Grafted cells survived and proliferated in the host brain, producing new neurons that migrated extensively and connected with host cells. We observed that reactive astrocytes were predominantly distributed close to xenografts, providing a migratory scaffold for graft-derived cells. Interestingly, in the striatum of hNSCs-treated mice, local astrocytes underwent de-differentiation, which might revert to the state of neuroepithelial precursor cells with multipotent differentiation potential for neurons or glia. Additionally, we also detected significantly higher expression of host-derived neurotrophic factors in hNSCs-treated mice compared with the control animals, together with attenuated activation of microglia and inhibition of proinflammatory cytokines, suggesting that hNSCs transplantation could alter host niche to provide neuroprotection.

### 3.1. Survival of Grafted hNSCs in the Striatum

Early studies reported that transplanted NSCs were able to survive and differentiate in PD rodents [6,7,8,25,26]. Only a small number of grafted cells were detectable, whereas approximately 75% dopaminergic neurons loss was observed after MPTP [8,26,27]. Yasuhara *et al.* [8] identified neurological improvement in response to hNSCs transplantation, with ~1% of grafts survival, little migration as well as differentiation into TH positive neurons. Harrower *et al.* [11] also reported poor survival and maturation of NSCs (~5%) even in immunosuppression PD rats. In our study, although cells within the xenografts continue to proliferate, few have been found to survive 42 days post-treatment. Moreover, limited hNSCs develop typically and express Dcx, the specific marker for migrating neuroblasts (Figure 4), suggesting that hNSCs have differentiated into new immature neurons.

### 3.2. Distribution of Local Astrocytes Response to hNSCs Transplantation

Astrocytes, the cells that compose the gliovascular interface, participate in regulation of blood flow, modulation of synaptic transmission, and nutrient transport such as L-dopa uptake [12,13]. These cells play a key role in the migratory process of endogenous NSCs/NPCs in adult animals. Migration along the RMS is characterized by chain migration in which neuroblasts migrate closely with each other in a tube-like structure established by astrocytic cells [5,14,28]. The specific glial tubes are critical in brain maturation, supporting the survival of immature neurons and facilitating their migration [28]. Previously, Uchida *et al.* [29] transplanted hNSCs into the lateral ventricle of neonatal mouse. They found the extent and pattern of cells migration were similar to endogenous NSCs/NPCs, which started from the subventricular zone (SVZ) to the olfactory bulb (OB). Indeed, our results support their findings that grafted hNSCs recruit host astrocytes to the grafted trajectory. Most grafted cells seem to form chains surrounded by these host-derived astrocytic structures, and migrate towards the cc as well as the RMS, which were mainly comprised of astrocytic cells (Figure 4H) (Figure 5A). We consider that hNSCs transplantation induce accumulation of local astrocytes which may provide the migratory scaffold for graft-derived cells.

In addition to distribution of local astrocytes, we also quantify the overall number of astrocytes residing in the striatum. Although there is a trend towards decrease of local astrocytes after hNSCs transplantation, we have found no significant difference between hNSCs-treated mice and the control animals. Previous reports demonstrated that neuroprotective effects were due to attenuated activation of astrocytes in PD models [6,30,31]. However, in the present study, significantly greater increase of reactive astrocytes undergoing proliferation has been revealed in mice receiving hNSCs. The response demonstrate that cell transplantation may recruit more reactive astrocytes to re-exhibit proliferative capacity.

### 3.3. EDAs as a Promising Source for Endogenous Repair

Astrocytes are the most abundant cells in the nervous system, which support neuronal survival and regeneration [32,33]. Reactive astrocytes, characterized by enlarged cell bodies, thick processes, and high expression of GFAP [34], is a prominent neuropathological feature, particularly in neurodegenerative disorders such as PD [35]. Evidence implicating neurodegeneration in the nigrostriatal pathway indicates that massive and prolonged reactive astrocytes are induced after exposure of MPTP in animals [23,36]. It appears that GFAP expression remains upregulated even after the main wave of dopaminergic structure destruction [37,38]. The precise effects exerted by the astrocytes in neurodegeneration and neuroregeneration processes are still highly controversial [31,36].

Reactive astrocytes were thought to play a minimal role in neuroprotection. However, an increasing body of literature suggests that these cells may turn into highly neuroprotective cells in certain circumstances [23,33,36]. They have been proposed as resident NSCs/NPCs in both the SVZ and striatum of adult animal [5]. A subset of GFAP positive astrocytic cells in the SVZ of rodents gives rise to transient amplifying cells, which then generate migrating neuroblasts [14,28]. Similarly, it is worth emphasizing that, reactive astrocytes in the striatum have also been proposed as proliferating NSCs/NPCs following injury [17,39].

In our study, we have demonstrated that the proliferating astrocytes in the striatum co-localize with both Nestin and Sox2, indicating that they have reverted to an immature state. Nestin represents a kind of intermediate filament and is expressed strongly in multipotential stem cells and precursor cells of the developing neuroepithelium [5,15,16]. It is downregulated when these cells differentiate into neuronal or glial cells both *in vivo* and *in vitro*. Lin *et al.* [15] showed re-expression of Nestin in reactive astrocytes which exhibit certain features of neuroepithelial cells, suggesting a recruitment of these cells from a pool of precursor cells. Wachter *et al.* [16] found reactive astrocytes co-expressed Ki-67, Nestin, and Pax6 in the striatum of PD rats. They considered these local cells derived from mature astrocytes that, upon dopaminergic denervation, became reactive and regained the ability of precursor cells, not from stem cell niches in the SVZ. The reversal was named de-differentiation, which transformed mature astrocytes into a phenotype resembling RGCs. In agreement with their interpretations, we have observed dramatically increased number of EDAs co-expressing Nestin and Sox2 in hNSCs-treated animals as well. Sox2 is a transcription factor involved in the development of the nervous system, which controls neuronal and astrocytic specification [5,16]. Early studies have shown that Sox2 is capable of reprogramming astrocytic cells to immature neurons [18,40]. The fact that the number of proliferating astrocytes which express Nestin as well as Sox2 is increased following transplantation has led us to speculate that xenografts to some extent induce and promote local astrocytes to de-differentiate and acquire the properties of NSCs/NPCs, which might further differentiate into a neuronal phenotype, such as neuroblasts or even neurons. The speculative idea may represent one of the possible compensatory mechanisms for improved behavioral functions following hNSCs transplantation in PD animals. Recently, investigators have reprogrammed reactive astrocytes into immature [18] or mature neurons [19] by transcription factors both *in vitro* and *in vivo*. Nevertheless, it remains uncertain whether the reactive astrocytes without being engineered *ex vivo* could definitely give rise to dopaminergic neurons in the DA-depleted striatum. Future studies shall attempt to reveal the reactive astrocytes response to hNSCs transplantation in the SN, and to corroborate our idea that EDAs could yield neuronal cells to exert neuroprotection.

### 3.4. Secretion of Neurotrophic Factors within Local Microenvironment

It is well known that survival of stem or precursor cells (both endogenous and exogenous cells) depends largely on their niche [10]. Harnessing the synergistic interaction between cells and niche may lead to the optimization of cell-based therapies for PD [6]. Importantly enough, the EDAs are known to release numerous neurotrophic factors including BDNF, NT-3, IGF-1, and GDNF both *in vivo* and *in vitro*, which effectively increase neurogenesis, promote neurite plasticity, and enhance functional recovery [7,20,21,41,42]. Based on the protective actions of neurotrophic factors on dopaminergic neurons [7,32,42,43], we examined host niche in the striatum and SN, using primers as well as antibodies specifically recognizing mouse but not human GDNF, BDNF, and NT-3. The hNSCs-treated group presented higher level of BDNF, NT-3, and GDNF than the control group at some time points. Motor improvement appeared in treated mice after seven days post-transplantation. Our findings support the concept that transplanted NSCs not only accommodate to their host but may stimulate their host to accommodate to them [7]. In other words, these cells can recruit EDAs, which may release growth factors to establish a more favorable niche, and a combination of both exogenous and endogenous cells can be extremely potent and advantageous.

Specifically, reactive astrocytes are the major source of GDNF after DA depletion [36,44,45]. Redmond *et al.* [46] considered that astrocytes highly expressed GDNF, and played a critical role in promoting effective processing and release of DA, which were consistent with previous findings. Notably, our results display that although the levels of GDNF in grafted animals are higher than controls, no significant difference is achieved at later stages. Several possible explanations can be elucidated here. Firstly, host-derived reactive astrocytes release an abundant supply of GDNF after DA depletion, determining baseline of this growth factor at high level in control mice. Secondly, we have demonstrated that animals in the control group underwent severe inflammatory response. Importantly, MPTP-induced inflammation produces a high amount of proinflammatory cytokines such as tumor necrosis factor-α (TNF-α) as well as interleukin-1β (IL-1β), which can elevate GDNF concentration [42,45]. Therefore, expression of GDNF in control group remains high at each time point.

In our study, we have revealed improvements in both the striatal and nigral tissues for neurotrophic factors even though the cells were transplanted in the striatum. It is well known that the nigrostriatal pathway, one of the major dopamine pathways in the brain, is made up of projections from SN to the striatum. Intriguingly, Sun *et al.* [47] found striatal dopaminergic projection neurons could form long axons that targeted the SN. Herzog *et al.* [48] clearly showed the pattern of retrograde transport of neurotrophic factors through the nigrostriatal axons in PD model. Neurotrophic factors were secreted from striatal cells, and retrograde transported to SN after uptake by nigrostriatal terminals. According to their findings, we speculate that the retrograde transport of neurotrophic factors leads to protection of residual dopaminergic neurons in the SN. Other possible explanations include regulating the host niche by local astrocytes in the SN, which needs to be testified.

### 3.5. Inhibition of Microglia and Proinflammatory Cytokines

Activation of microglia is the feature of neuroinflammation in PD [23,24]. Notably, the decrease of activated microglia was formerly thought to parallel the inhibition of astrocytes following treatment [30,49]. Nevertheless, as previously discussed, our data show that, although there is no difference in the number of striatal astrocytes between mice given hNSCs and vehicle, fewer microglia are observed in hNSCs-treated mice. We have found that most microglia are distributed around the xenografts, suggesting that the hNSCs could gather microglia to a limited area and thus attenuate overall inflammation in the brain niche. Additionally, we have revealed a significant downregulation of proinflammatory cytokines, such as IL-1β and TNF-α. Consequently, we consider that hNSCs induce anti-inflammatory effects on the host niche of Parkinsonian mice.

## 4. Experimental Section

### 4.1. Preparation of hNSCs

GFP-labeled human neural stem cell line was obtained from a neural stem cell line deriving from human fetal brain (Angecon Biotech, Shanghai, China) by lentivirus-mediated gene transfer to express GFP, and prepared for transplantation. All procedures were approved by the Human Experimentation and Ethics Committee. Briefly, primary dissociated single cell suspensions from human cortex tissues of legally terminated embryos with approval of National Health and Family Planning Commission of the People’s Republic of China, were incubated in serum-free NSC medium (Angecon Biotech, Shanghai, China) according to the manufacturer’s instruction. A plating density of 100 cells/μL favored the establishment of neurospheres at 37 °C with 5% CO_2_. Neurosphere cultures were digested with 0.05% trypsin-EDTA (Invitrogen, Carlsbad, CA, USA) followed by trypsin inhibitor (Roche, Mannheim, Baden-Württemberg, Germany), and were replated at 100 cells/μL every 7 to 10 days. On the day of transplantation, hNSCs were dissociated and rewashed before a final concentration adjustment.

### 4.2. MPTP Administration

All animal experiments were performed in accordance with guidelines on the care of laboratory animals, and have been approved by the ethics committee of Peking Union Medical College Hospital. Adult C57BL/6J female mice (10-week-old) weighing approximate 25 g were maintained in a 12-h light/dark circle in cages, and acclimated to the experimental environment for 1 week before modeling. PD model was induced by acute MPTP exposure [27]. MPTP hydrochloride (20 mg/kg; Sigma, St. Louis, MO, USA) in saline was injected intraperitoneally four times in 1 day at 3 h intervals. MPTP was handled in accordance with the published guideline [27].

### 4.3. Behavioral Tests

#### 4.3.1. Rotarod

Rotarod test was performed on day 7, 14, 21, 28, 35, 42, 49 after MPTP injection. All mice were pre-trained about 1 week before tests with rod diameter of approximate 3 cm. The training consisted of 3 consecutive runs at 26 rpm until the mice were unable to maintain themselves for 300 s on the rotating rod and lasted for 3 days. One day prior to MPTP administration, baseline was obtained under the same condition. Subsequently, test series started at speed of 26 rpm. Each animal received 2 trials with a rest interval of 5–10 min, and the latencies to fall off the rod within 180 s were recorded and averaged.

#### 4.3.2. Pole Test

Mice were placed head upwards on the top of one upright pole approximate 30 cm long as well as 8 mm in diameter. The base of the pole was maintained in the cage. Once laid upon the top, mice oriented themselves downwards and descended to the ground. Animals also received 3 days training which consisted of 3 consecutive trials of each session. After establishing the baseline, each animal experienced 2 trials and the time to descend was recorded.

### 4.4. Cell Transplantation

Mice subjected to MPTP were randomly assigned to two groups: (1) the hNSCs-treated group (MPTP + hNSCs), and (2) the control group (MPTP + vehicle). Mice received bilaterally intrastriatal transplantation of undifferentiated hNSCs or vehicle 7 days after MPTP administration. Following anesthesia, animals were secured onto the stereotactic frame (Stoelting, Wood Dale, IL, USA) using an incisor clamp and two ear bars. A midline rostro-caudal incision was created and then, two burr holes were formed bilaterally at the sites based on coordinates relative to bregma (left, anteroposterior, +0.06 cm, mediolateral, +0.20 cm; right, anteroposterior, +0.06 cm, mediolateral, −0.20 cm) by a high-speed drill. Each animal in treated group received 2 μL of cell suspension bilaterally at coordinates: dorsoventral, −0.22 and −0.28 cm, relative to dura. Approximately 5 × 10^4^ hNSCs in 0.5 μL PBS or an equal volume of vehicle was transplanted at 4 sites by microsyringe at an infusion rate of 0.1 μL/min for a total dose of 2 × 10^5^/2 μL. The needle was withdrawn slowly following a wait of 2 min. Animals were returned to a temperature-controlled blanket until they recovered from anesthesia.

### 4.5. Fixation and Immunohistochemistry

To determine transplanted cell survival, neuronal phenotype expression, and synapse formation, immunohistochemical procedures were conducted. At day 7, 14, 28, 42 after cell engraftment, under deep anesthesia, animals were perfused intracardially with cold 0.1 M PBS followed by 4% paraformaldehyde (PFA). Brains were carefully removed and post-fixed at 4 °C overnight, cryoprotected in 30% sucrose for 3 days. A complete set of coronal sections containing the striatum as well as the SN were then created at a thickness of 30 μm using freezing microtome (Leica, Nussloch, Baden-Württemberg, Germany) and stored at −20 °C. Every eighth section of striatum or sixth section of SN was processed.

#### 4.5.1. Immunofluorescence Staining

Primary antibody of GFP (1:500, Aves, Tigard, OR, USA) was used to identify transplanted hNSCs. Brain slices were processed free-floating at 4 °C overnight with specific primary antibodies, diluted in 0.1 M PBS containing 0.3% Triton X-100 and 5% Bovine Serum Albumin (BSA) (Sigma-Aldrich, St. Louis, MO, USA). To detect co-localization of cell type-specific markers, each slice was incubated with Sox2 (1:100, Millipore, Billerica, MA, USA), Nestin (1:500, Millipore, Billerica, MA, USA), Ki-67 (1:200, Thermo Fisher Scientific, Waltham, MA, USA), Dcx (1:200, Santa Cruz Biotechnology, Santa Cruz, CA, USA), and GFAP (1:600, Millipore, Billerica, MA, USA) respectively. All the antibodies were tested on the brain tissues including positive and negative controls to assure their specificity. Secondary antibodies (all from Invitrogen, Life technologies, Carlsbad, CA, USA) in 0.1 M PBS containing 0.3% Triton X-100 and 5% BSA were utilized and listed: Alexa Fluor^®^ 488 donkey anti-mouse (1:1000), Alexa Fluor^®^ 488 donkey anti-chicken (1:1000), Alexa Fluor^®^ 546 donkey anti-goat (1:1000), Alexa Fluor^®^ 594 goat anti-chicken (1:1000), Alexa Fluor^®^ 647 donkey anti-mouse (1:1000), Alexa Fluor^®^ 647 donkey anti-rabbit (1:1000). All staining procedures were conducted in the dark room for 2 h at room temperature in combination with nucleus counterstaining 4′,6-diamidino-2-phenylindole (DAPI, 0.1 µg/mL, Sigma-Aldrich, St. Louis, MO, USA). Finally, sections were washed with 0.1 M PBS, mounted on glass slides and coverslipped with medium.

#### 4.5.2. Peroxidase Immunohistochemistry

Immunoperoxidase staining for TH, GFAP, and IBA-1 were performed. Brain slices were processed free floating, pretreated for peroxidase activity using 3% hydrogen peroxide (H_2_O_2_) in 0.1 M PBS containing 5% Triton X-100 for 20 min, and blocked with 5% normal horse serum (NHS) (Vector laboratories, Burlingame, CA, USA) for 1 h. Thereafter, selected tissue slices were incubated with primary antibodies of TH (1:800, Millipore, Billerica, MA, USA), GFAP (1:600, Millipore, Billerica, MA, USA), and IBA-1 (1:400, Wako, Chūō-ku, Osaka, Japan) at 4 °C overnight. Slices were again washed 3 times with 0.1 M PBS, incubated with biotinylated horse anti-rabbit IgG (Vector laboratories, Burlingame, CA, USA) or goat anti-chicken IgG (Vector laboratories, Burlingame, CA, USA) secondary antibody for 1 h, and then treated with biotinylated protein A and avidinbiotinylated horseradish peroxidase complexes (ABC kit, Vector laboratories, Burlingame, CA, USA) for another 1 h at room temperature. Immunoreactive cells were visualized with 3,3′-diaminobenzidine (DAB) and urea H_2_O_2_ tablets (Sigma-Aldrich, St. Louis, MO, USA) dissolved in double distilled water. As negative controls, immunohistochemistry was performed without the primary antibodies. Following immunoperoxidase staining, sections were hematoxylin counterstained. Finally, sections were mounted onto glass slides, dehydrated through a graded series of ethanol, cleared in xylene, and coverslipped.

### 4.6. QRT-PCR Analysis of Gene Expression in the Striatum and VM

QRT-PCR was preformed on mRNA from striatum and ventral mesencephalon (VM) mainly containing the SN to analyze the expression of GDNF, BDNF, and NT-3. Total RNA was extracted from different tissues using Trizol reagent (Invitrogen, Life technologies, Carlsbad, CA, USA) according to the recommendations of the manufacturer. RNA concentration was determined by the spectrophotometric measurement of 260 nm with NanoDrop2000/2000c (Thermo Fisher Scientific, Waltham, MA, USA). Using cDNA reverse transcription kit (Applied Biosystems, Life technologies, Carlsbad, CA, USA), two micrograms of RNA were reverse transcribed. Two microlitres of reverse transcribed cDNA was amplified in a 20 μL of reaction mixture containing 10 μL SYBR^®^ Premix Ex TaqTM (Takara Bio, Otsu, Shiga, Japan) and 0.5 μL of each primer. The amplified procedure consisted of 95 °C for 5 min, and 40 cycles of reactions at 95 °C for 10 s, 60 °C for 30 s. The primers were as follows: GDNF (82 bp), forward 5′-TCCAACTGGGGGTCTACGG-3′, reverse 5′-GCCACGACATCCCATAACTTCAT-3′; BDNF (137 bp), forward 5′-TCATACTTCGGTTGCATGAAGG-3′, reverse 5′-AGACCTCTCGAACCTGCCC-3′; NT-3 (96 bp), forward 5′-AACAGAGACGCTACAATTCGC-3′, reverse 5′-GGTTGCCCACATAATCCTCCAT-3′; GAPDH, (133 bp), forward 5′-ACAACTTTGGCATTGTGGAA-3′, reverse 5′-GATGCAGGGATGATGTTCTG-3′. cDNA of GAPDH was used as an internal control. Relative expression of a given sample was normalized to an internal control, and was calculated using the 2^−∆∆*C*t^ method.

### 4.7. ELISA Analysis

Mice were sacrificed via cervical dislocation, and tissues of striatum and VM were collected respectively. Homogenized samples with high protein content were subjected to mouse ELISA kit (Abcam, Cambridge, Cambridgeshire, UK) to determine protein concentration of BDNF, GDNF, NT-3, IL-1β, and TNF-α. Each assay was performed in accordance with manufacturer’s instructions. The concentrations in the brain tissues were expressed as pg/mL (BDNF, GDNF, IL-1β, and TNF-α) or ng/mL (NT-3) total protein.

### 4.8. Quantification

Fluorescently immuno-labeled sections were analyzed on a Zeiss LSM780 confocal laser-scanning microscope (Carl Zeiss, Oberkochen, Baden-Württemberg, Germany), and images were captured and reconstructed using the Zeiss LSM software. Immunoreactive cells were analyzed with Bitplane-Imaris software 7.2 (Bitplane, Belfast, County Antrim, UK) manually under blinded conditions on coded slides. Briefly, utilizing 20× lens, 6 regions (3 on the left hemisphere and 3 on the right) were evaluated bilaterally in 3 representative sections including the grafted cells (0.73, 0.43, 0.13 mm anterior to the bregma, 30-μm thick, 300-μm interval) in each animal. Grafted cells in the striatum were counted in 4 representative animals per group [6]. Data were expressed as the mean ± SEM of the percent of GFP positive cells co-labeling with specific markers. Grafts survival was estimated as percentage of GFP-expressing cells at each time point compared with day-7 group.

Nonfluorescent immuno-stained sections were captured on a Nikon Eclipse 90i microscope (Nikon, Shinagawa, Tokyo, Japan) and analysis was performed using the Stereo Investigator (MicroBrightField Bioscience, Williston, VT, USA) as previously described [50]. For each animal, GFAP and IBA-1 positive cells (astrycytes and microglia) in the striatum and cells expressing TH (Dopaminergic neurons) in the SN were analyzed. The entire striatum or SN was identified as region of interest for each section. The number of immunoreactive cells in each counting frame (150 μm × 150 μm) was then analyzed under 20× objective using optical dissector method. Optical dissectors were 150 μm × 150 μm × 25 μm cubes spaced in a systematic random manner 300 μm × 300 μm apart and offset 4 μm from the section surface. A positive cell was defined as the presence of its nucleus either within the counting frame or touching green frame lines, but not touching red frame lines. The total number of positive cells in the striatum was calculated as raw counts × 8 (section evaluation) × 4 (area of grid 300 μm × 300 μm divided by area of counting frame 150 μm × 150 μm) × 25 μm (average mounted thickness)/17 μm (optical dissector height). For sections containing SN, total number equaled raw counts × 6 (section evaluation) × 4 × 25 μm/17 μm. The optical densities of striatal TH-immunostained sections were analyzed with ImageJ (NIH, Bethesda, MD, USA).

### 4.9. Statistical Analysis

All data were presented as mean ± SEM and analyzed using GraphPad Prism 6.0 (GraphPad Software, San Diego, CA, USA) for Mac OS X. The difference between two groups was analyzed by a two-tailed Student’s *t*-test, while two-way ANOVA followed by Tukey’s post-hoc analysis were conducted for multiple comparisons between two or more groups. A *p* value of less than 0.05 was defined as a threshold for statistical significance.

## 5. Conclusions

hNSC transplantation provides cues for recruiting local astrocytes to the grafted area, stimulating de-differentiation of host-derived astrocytes, followed by niche regulation including production of endogenous growth factors as well as attenuation of microglia activation. Both the grafts and host cells within the brain niche can interact to achieve neuroprotection against DA depletion. Harnessing synergistic interactions may help optimize cell-based therapies for PD.
